# A Case of Congenital Peritoneal Encapsulation Complicated with Left Paraduodenal Hernia Presenting as Recurrent Small Bowel Obstruction

**DOI:** 10.70352/scrj.cr.26-0368

**Published:** 2026-07-14

**Authors:** Nodoka Tominaga, Kaido Oishi, Hiroaki Takeshita, Tetsuhiro Tomiyama, Narumi Ota, Tsubasa Kubo, Saeko Fukui, Mampei Yamashita, Tota Kugiyama, Takanobu Hara, Michi Morita, Mio Fujii, Akira Yoneda, Shigeki Minami, Tamotsu Kuroki

**Affiliations:** Department of Surgery, NHO Nagasaki Medical Center, Omura, Nagasaki, Japan

**Keywords:** congenital peritoneal encapsulation, left paraduodenal hernia, small bowel obstruction, strictureplasty, postoperative adhesion, laparoscopy

## Abstract

**INTRODUCTION:**

Congenital peritoneal encapsulation (CPE) is a rare anomaly in which the small intestine is enclosed in a thin peritoneal sac. Although mostly asymptomatic, CPE can cause severe intestinal obstruction due to strangulation and torsion.

**CASE PRESENTATION:**

A 16-year-old male patient had been experiencing recurrent intestinal obstruction of unknown cause since the age of 13. He was admitted with persistent abdominal pain as his chief complaint, and emergency laparoscopic surgery was performed due to a suspected strangulating intestinal obstruction. The findings included an abnormal course of the inferior mesenteric vein (IMV), severe intussusception of the small intestine into the descending mesentery, ileal adhesions, and a segment of the small intestine covered by a thin membrane. The patient was diagnosed with CPE and a left paraduodenal hernia. Initially, the hernia was treated with manual reduction, and CPE was managed by excising the accessory peritoneal membrane and performing adhesiolysis. However, recurrent obstruction occurred due to dense terminal ileal readhesion, necessitating a reoperation on the 21st POD. During reoperation, side-to-side strictureplasty was performed for the adhesion, and the paraduodenal hernia was definitively managed by unroofing the hernia sac. The patient recovered uneventfully, without recurrence.

**CONCLUSIONS:**

Complex congenital anatomical abnormalities, such as CPE and internal hernias, should be considered in recurrent intestinal obstruction from childhood to young adulthood. If CPE is confirmed intraoperatively, investigation of other congenital anomalies is warranted, and the possibility of robust postoperative adhesions due to congenital fusion planes must be considered.

## Abbreviations

CPE
: congenital peritoneal encapsulation
CRP
: C-reactive protein
IMV
: inferior mesenteric vein
LCA
: left colic artery
SBO
: small bowel obstruction
SEP
: sclerosing encapsulating peritonitis
SIBO
: small intestinal bacterial overgrowth
SMV
: superior mesenteric vein

## INTRODUCTION

CPE, first reported by Cleland in 1868, is a rare anomaly in which part or all of the small intestine is encapsulated by a thin, transparent, accessory peritoneal membrane.^1)^ Its embryological origin is linked to anomalies during midgut rotation and the reduction of the physiological umbilical hernia into the abdominal cavity, usually around the 10th to 12th week of gestation.^2)^ It is hypothesized that the yolk sac-derived peritoneum, which should regress, persists and forms an anomalous membrane around the small bowel loops.

Clinically, because the peristaltic function of the encapsulated bowel is generally preserved, most patients remain asymptomatic. It is often discovered incidentally during autopsies or laparotomies for unrelated pathologies.^3–5)^ However, CPE can rarely cause mechanical SBO due to volvulus or strangulation at the membrane’s opening or compression by fibrous bands, requiring urgent surgery.^6–12)^ CPE lacks specific symptoms and often eludes preoperative imaging diagnosis, making it a potentially overlooked cause of acute abdomen. The standard surgical approach for CPE-induced bowel obstruction involves excising the accessory membrane and performing adhesiolysis of inter-intestinal adhesions.^2,9)^

This paper reports a case of CPE in combination with a left paraduodenal hernia, an extremely rare congenital anomaly, accompanied by a review of the literature.

## CASE PRESENTATION

Case: A 16-year-old male.

Chief complaint: Persistent worsening of abdominal pain since the previous day. Past medical history: The patient had idiopathic intestinal obstruction at the ages of 13 and 15 years, which was resolved with conservative treatment. No abnormalities were found in thorough examinations, including lower gastrointestinal endoscopy at age 15, and no clear cause was identified. Present illness: The patient was transported to our hospital via emergency services due to abdominal pain persisting since the previous day and worsening.

**Fig. 1:**
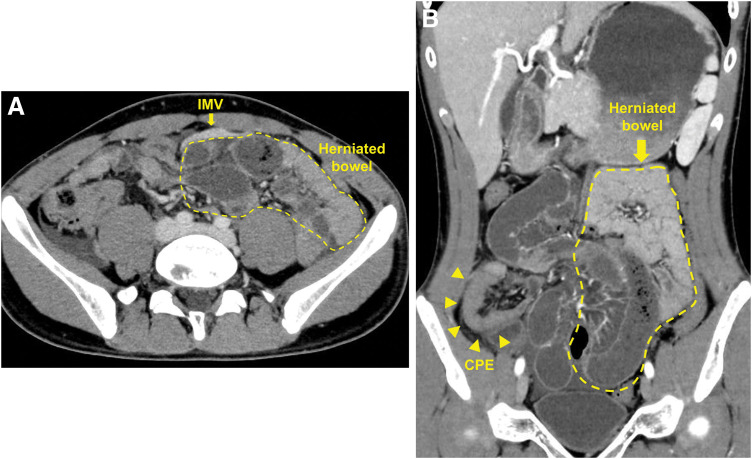
Preoperative contrast-enhanced CT findings. (**A**) Axial view. A partially dilated loop of the small intestine was observed herniating into the left side of the abdomen and pelvis (area enclosed by dashed lines), positioned dorsally to the abnormally routed the IMV (indicated by the arrow). These findings are indicative of left paraduodenal hernias. (**B**) Coronal view. The complex pathology is depicted. Another segment of the small intestinal loop is encased within a thin accessory peritoneum (indicated by arrowheads) in the right lower abdomen, a finding consistent with CPE. Additionally, a large hernia was observed in the left paraduodenal space. CPE, congenital peritoneal encapsulation; IMV, inferior mesenteric vein

Physical examination: Mild abdominal distension and tenderness, with no signs of peritoneal irritation. Laboratory findings: White blood cell count was 15400/μL, and the CRP level was 0.018 mg/dL, indicating mild inflammation. Imaging findings: Contrast-enhanced abdominal CT showed massive herniation of the small bowel loops, dorsal to the descending mesocolon, with marked dilation, multiple caliber changes, and closed-loop formation. The terminal ileum was clustered into a mass in the right lower abdomen (**Fig. 1**). Based on these findings, a diagnosis of strangulated SBO due to a left paraduodenal hernia was made, and emergency laparoscopic surgery was performed.

**Fig. 2:**
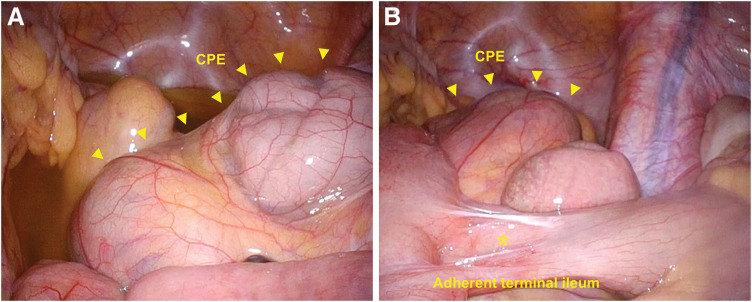
Intraoperative findings of CPE. (**A**) Laparoscopic view. The cluster of small intestinal loops was completely enclosed by a thin, transparent, accessory peritoneum (indicated by arrowheads). (**B**) The terminal ileum is strongly adhered to and twisted (indicated by an asterisk). The small intestinal loops passing through this area are enclosed by the accessory peritoneum (indicated by arrowheads). This illustrates the anatomical relationship between the neck and the CPE sac. CPE, congenital peritoneal encapsulation

Laparoscopy revealed the presence of serous ascites; however, no irreversible ischemic changes, such as frank intestinal strangulation or necrosis, were detected. A substantial loop of the small intestine, beginning from the proximal jejunum, herniated into the dorsal side of the descending mesocolon (Landzert’s fossa) behind the IMV, establishing a diagnosis of left paraduodenal hernia. The terminal ileum was firmly adherent to the proximal ileal loop with a pronounced twist. Additionally, the segments of the small intestine between the adhesions were encased by a thin accessory peritoneum, with no interloop adhesions observed within this enclosed segment, which is characteristic of CPE (**Fig 2**). Given the urgent nature of the surgical intervention and the necessity to prevent vascular injury, the treatment for the left paraduodenal hernia was limited to manual reduction of the small intestine from Landzert’s fossa. Definitive repair or closure of the hernia orifice was deferred during this initial emergency operation primarily because the severely dilated bowel loops significantly restricted the laparoscopic surgical field, making any suturing maneuver hazardous, with a high risk of iatrogenic vascular injury. Furthermore, because there were no findings of severe stricture or frank strangulation compromising bowel viability, we concluded that further invasive intervention was unnecessary at this acute stage, and prioritizing a safe, minimal procedure was the most appropriate strategy. For the management of CPE, surgical intervention involved dissection and release of strong adhesions between the ileal loops at the membrane attachment, followed by resection of the accessory peritoneum. No adhesion prevention agents were used after the initial surgery.

**Fig. 3:**
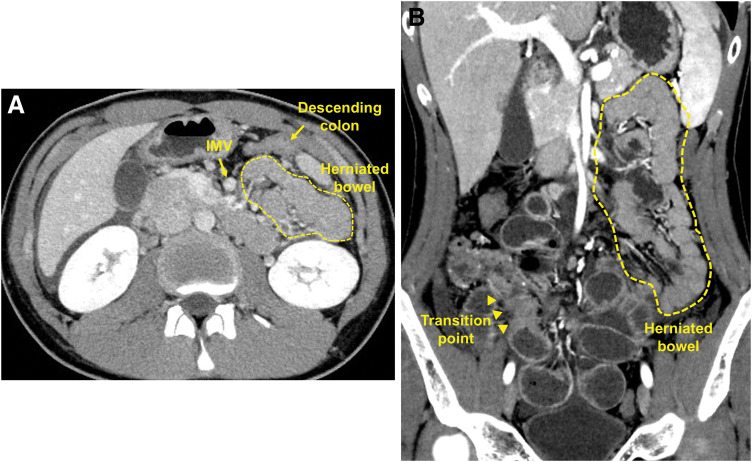
CT findings at the time of readmission. (**A**) Axial view. A segment of the small intestine was observed herniating into Landzert’s fossa (area enclosed by dashed lines and labeled “Herniated bowel”). The IMV coursing ventrally to the herniated loops and descending colon is indicated by arrows. (**B**) Coronal view. The concurrent recurrence of the left paraduodenal hernia is depicted (area enclosed by dashed lines and labeled “Herniated bowel”). The transition point in the terminal ileum, which represents the twisted adhesion and the primary cause of recurrent obstruction, is also shown (indicated by arrowheads). Note that marked bowel dilation within the hernia sac is not prominent, consistent with the clinical finding that the primary obstruction was at the terminal ileum. IMV, inferior mesenteric vein

**Fig. 4:**
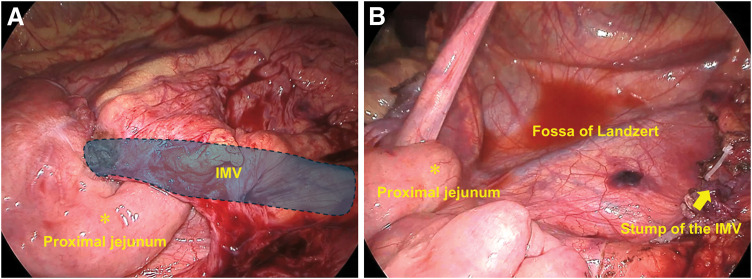
Intraoperative findings of a left paraduodenal hernia during reoperation. (**A**) Image after reduction of the herniated intestine. The IMV (highlighted in blue) crossed the proximal jejunum (indicated by an asterisk) from the ventral side, forming the anterior edge of the hernia opening. (**B**) The image shows an opened hernia sac. The descending mesentery was incised following sacrificial ligation and transection of the IMV and LCA. This procedure effectively reduced the large non-physiological space of Landzert’s fossa (indicated by the label “Fossa of Landzert”). IMV, inferior mesenteric vein; LCA, left colic artery

The patient was discharged on POD 7 but developed recurrent abdominal pain and was readmitted on day 10. Contrast-enhanced CT scan at readmission demonstrated dilation of the small intestine with clustering and complex twisting at the terminal ileum, indicating adhesive SBO as the primary cause of recurrent ileus. Concurrently, although marked bowel dilation within the hernia sac was absent, herniation of the small bowel loops dorsal to the IMV was clearly observed, confirming the recurrence of the left paraduodenal hernia (**Fig. 3**). Conservative management failed, and reoperation was performed on day 21. Intraoperative examination revealed a recurrence of the left paraduodenal hernia, accompanied by significant readhesion of the terminal ileum. A substantial segment of the small intestine was observed to protrude into Landzert’s fossa, indicating that the left paraduodenal hernia likely contributed to the recurrent bowel obstruction experienced before the initial surgical intervention. Furthermore, upon detailed exploration during reoperation, a rare anatomical anomaly of the vasculature was definitively identified; the IMV was observed coursing ventral to the ligament of Treitz and draining directly into the SMV. Due to this anomalous course, the proximal jejunum was located within the hernial sac (**Fig. 4A**).

**Fig. 5:**
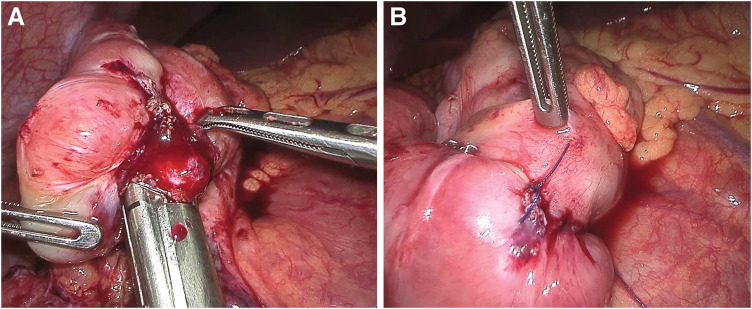
Intraoperative photographs of laparoscopic strictureplasty. (**A**) A small enterotomy was created at the apex of the U-shaped kinked terminal ileum. The 2 jaws of an endoscopic linear stapler are shown inserted through this single hole into the proximal and distal limbs and are ready to be fired. (**B**) Completed functional side-to-side bypass. The common enterotomy was securely closed with a continuous barbed suture, effectively resolving the stricture while preserving the ileocecal region.

Moreover, Landzert’s fossa was exceptionally large. Based on these precise anatomical evaluations, we determined that simple closure of the hernia orifice would be highly hazardous, posing a risk of proximal jejunal stricture or leaving a massive postoperative dead space. To address this anatomical anomaly definitively, after confirming adequate blood flow in the marginal arteries and veins, the LCA and IMV, which formed the hernial orifice, were dissected and transected. The mesentery of the descending colon, which formed the hernia sac, was then partially resected to unroof and completely open the hernia sac (**Fig. 4B**). The readhesion responsible for the obstruction was located 5–10 cm from the terminal ileum, where the intestinal loop was acutely kinked into a U-shape and firmly adhered to the proximal small intestine. Complete dissection and straightening of the bowel were extremely challenging because of the robust adhesions. Although resection of the ileocecal region was considered, we opted for laparoscopic strictureplasty to avoid iatrogenic bowel injury and to preserve the ileocecal valve. Technically, a small enterotomy was created at the apex of the U-shaped folded loop. A small enterotomy was created at the apex of the U-shaped folded loop. The 2 jaws of an endoscopic linear stapler (Signia purple 45; Medtronic, Minneapolis, MN, USA) were inserted through this single hole, one placed into the proximal limb and the other into the distal limb of the kinked ileum (**Fig. 5A**). Firing the stapler created a wide functional side-to-side bypass, successfully achieving strictureplasty. The common enterotomy was closed using full-thickness continuous sutures with a 3-0 unidirectional barbed suture (Stratafix Symmetric PDS Plus; Ethicon, Somerville, NJ, USA) (**Fig. 5B**). A spray-type adhesion barrier (Adspray; Terumo, Tokyo, Japan) was applied to prevent future readhesions.

The patient’s postoperative course after the second surgery was uneventful. Oral intake began on the third POD, and the patient was discharged on the seventh POD. Two months postoperatively, no abdominal pain or recurrence of intestinal obstruction was observed.

## DISCUSSION

This case illustrates a rare pathology resulting from an embryological anomaly, where the small intestine is enclosed in a congenital membrane, causing recurrent bowel obstruction. Differentiating it from similar diseases like SEP and abdominal cocoon syndrome is vital for treatment. Abdominal cocoon syndrome is acquired from chronic peritoneal inflammation, often due to dialysis or peritonitis, forming a fibrous capsule with dense intestinal adhesions.^4,8,13)^ In contrast, CPE is a congenital condition. Embryologically, the accessory peritoneal membrane is believed to be derived from the yolk sac-derived peritoneum. During the normal reduction of the physiological umbilical hernia into the abdominal cavity around the 10th to 12th week of gestation, abnormal adherence or incomplete regression causes the yolk sac peritoneum to be drawn into the peritoneal cavity, creating an extra-peritoneal sac that envelopes the small intestines. This results in an independent, thin, translucent membrane.^4)^ Histologically, unlike the fibrotic capsule seen in acquired SEP, this accessory membrane is identical to the normal visceral and parietal peritoneum. The key diagnostic feature is the lack of inflammatory adhesions among encapsulated intestines, which maintain bowel segment independence.^2,8,13)^ In this case, the gross intraoperative findings—specifically the thin, transparent nature of the sac and the complete absence of interloop adhesions (**Fig. 2**)—definitively confirmed a CPE diagnosis, excluding abdominal cocoon pathology. Because these macroscopic features are clinically decisive and sufficient for differential diagnosis, formal histological evaluation of the excised membrane was not performed.

CPE is often asymptomatic unless it causes bowel obstruction, complicating the accurate prevalence and incidence rates.^2,8)^ It is extremely rare; since 1868, approximately 50 cases have been reported in the English medical literature.^1,3)^ As a congenital disease, it may cause SBO in childhood or adolescence,^8,14)^ but can remain latent for decades, presenting at an advanced age, such as 82 years.^7)^ Therefore, it should be considered in the differential diagnoses for acute abdomen across all ages.

Because the development of this accessory membrane is closely linked to midgut reduction, CPE is frequently associated with high rates of concurrent congenital gastrointestinal anomalies.^2)^ The literature notes cases with intestinal malrotation,^15)^ incomplete situs inversus, Meckel’s diverticulum with strangulation,^6)^ or vascular anomalies causing SMV thrombosis.^13)^ In this case, although the colon’s position and retroperitoneal fixation seemed intact, exploration showed Toldt’s fascia fusion failure in the descending mesocolon and a rare vascular anomaly with the IMV draining into the SMV, provoking a giant left para-duodenal hernia. While Wani et al. reported obstruction with CPE, a left paraduodenal hernia, and IMV retroperitonealization,^16)^ such complex anatomical anomalies are rare. When the accessory peritoneum is found during surgery, resolving ileus by membrane excision is vital. Surgeons should suspect anomalies like intestinal malrotation, Meckel's diverticulum, or internal hernias and explore the abdominal cavity to prevent complications.

In CPE-induced bowel obstruction surgery, complete accessory membrane excision ensures a favorable prognosis.^9,10)^ However, postoperative adhesive SBO can occur. Wolski et al. documented a case needing reintervention for SBO due to adhesions after CPE excision in a 12-year-old.^14)^ Reports suggest postoperative peritonitis from excessive local inflammation after membrane excision.^7)^ In the present case, the terminal ileum formed dense readhesions approximately a week after surgery, causing obstruction. The absence of adhesion prevention agents during the initial emergency surgery likely exacerbated the severity of these early postoperative adhesions. The attachment sites of the accessory peritoneum, particularly the neck of the sac, consist of robust congenital and anatomical fusions. Previous reports have emphasized the need for extreme caution during dissection of these areas to completely liberate the intestine and avoid bowel injury.^2,17,18)^ However, as observed in our case, the extensive serosal and peritoneal defects inevitably resulting from this challenging dissection, inherently constitute a high-risk zone for severe postoperative readhesions. Therefore, while completely freeing the intestine at the entry and exit points of the CPE is a crucial step, surgeons must concurrently recognize the anatomical inevitability of early readhesion at these sites and proactively implement preventive measures, such as the use of adhesion barriers.

During reoperation, forceful adhesiolysis was avoided to prevent extensive serosal injury or perforation necessitating ileocecal resection, particularly because the terminal ileum was securely fixed in a U-shaped configuration by dense readhesions. In young patients, loss of the ileocecal region leads to significant malabsorption of bile acids and vitamin B12,^19)^ along with chronic diarrhea and SIBO due to the loss of Bauhin’s valve. Therefore, we opted for laparoscopic strictureplasty using an automated stapler by inserting the device through the apex of the kinked bowel to create a functional side-to-side bypass. This safely bypassed the stricture while preserving intestinal length and valvular function. This bowel-sparing approach is particularly effective in pediatric surgery, where long-term QOL and growth are paramount.^19–21)^

Proper management of left paraduodenal hernias is crucial to avoid these complications. We presumed that the preoperative recurrent obstructions were associated with both CPE-related adhesions and internal herniation. While the standard surgical approach for a left paraduodenal hernia is the closure of the hernia orifice, we encountered an exceptionally large Landzert’s fossa in this case. We presumed that simple closure would leave a massive dead space, posing potential risks for fluid accumulation, subsequent infection, and even hernia recurrence. Furthermore, as the IMV coursed ventrally to the ligament of Treitz, the proximal jejunum was located within the hernia sac. Under these anatomical conditions, any attempt to close the orifice—even a partial closure on the left side of the IMV to preserve the vessels—could potentially lead to stricture or kinking of the proximal jejunum, or result in incomplete closure of the hernia. These anatomical complexities and the associated risks strongly justified our decision to defer the definitive closure of the hernia orifice during the initial emergency operation, where severe bowel dilation severely limited the surgical field, regardless of whether a laparoscopic or open surgical approach was used. Although major vessel division is generally avoided in young patients, we confirmed adequate collateral blood flow through the marginal vessels. Consequently, we ligated the IMV and LCA and unroofed the descending mesocolon. This proactive approach eliminates non-physiological space and offers a definitive therapeutic option to prevent dead space-related complications.

## CONCLUSIONS

CPE should be considered in the differential diagnosis of bowel obstruction in patients without a surgical history, particularly during childhood. Given its association with other congenital anomalies, thorough intraoperative exploration is imperative when CPE is identified. While the fundamental treatment is complete excision of the accessory peritoneal membrane, surgeons must recognize that congenital fusion planes at the membrane's attachment sites carry a high risk of severe postoperative readhesions. Therefore, meticulous surgical handling and proactive use of prophylactic adhesion barriers are essential. Furthermore, in managing these complex readhesions or strictures, facile bowel resection should be avoided to prevent the long-term deterioration of the patient's QOL. Instead, a flexible surgical strategy prioritizing organ preservation, such as strictureplasty, is recommended.

## DECLARATIONS

### Availability of data and materials

The data that support the findings of this study are available from the corresponding author upon reasonable request.
